# A ferredoxin-dependent dihydropyrimidine dehydrogenase in *Clostridium chromiireducens*

**DOI:** 10.1042/BSR20201642

**Published:** 2020-07-02

**Authors:** Feifei Wang, Yifeng Wei, Qiang Lu, Ee Lui Ang, Huimin Zhao, Yan Zhang

**Affiliations:** 1 Tianjin Key Laboratory for Modern Drug Delivery and High-Efficiency, Collaborative Innovation Center of Chemical Science and Engineering, School of Pharmaceutical Science and Technology, Tianjin University, Tianjin 300072, China; 2 Frontiers Science Center for Synthetic Biology (Ministry of Education), Tianjin University, Tianjin 300072, China; 3 Singapore Institute of Food and Biotechnology Innovation, Agency for Science, Technology and Research (A*STAR), Singapore; 4 Department of Chemical and Biomolecular Engineering, University of Illinois at Urbana-Champaign, 600 South Mathews Avenue, Urbana, IL 61801, U.S.A.

**Keywords:** clostridium, Dihydropyrimidine dehydrogenase, Fe-S cluster, ferredoxin, Pyrimidine, reductive pyrimidine degradation

## Abstract

Dihydropyrimidine dehydrogenase (PydA) catalyzes the first step of the reductive pyrimidine degradation (Pyd) pathway in bacteria and eukaryotes, enabling pyrimidines to be utilized as substrates for growth. PydA homologs studied to date catalyze the reduction of uracil to dihydrouracil, coupled to the oxidation of NAD(P)H. Uracil reduction occurs at a flavin mononucleotide (FMN) site, and NAD(P)H oxidation occurs at a flavin adenine dinucleotide (FAD) site, with two ferredoxin domains thought to mediate inter-site electron transfer. Here, we report the biochemical characterization of a Clostridial PydA homolog (PydAc) from a Pyd gene cluster in the strict anaerobic bacterium *Clostridium chromiireducens*. PydAc lacks the FAD domain, and instead is able to catalyze uracil reduction using reduced methyl viologen or reduced ferredoxin as the electron source. Homologs of PydAc are present in Pyd gene clusters in many strict anaerobic bacteria, which use reduced ferredoxin as an intermediate in their energy metabolism.

## Introduction

The reductive pyrimidine degradation (Pyd) pathway is the most widespread pathway for the degradation of the pyrimidine ring, and is present in bacteria, archaea and eukaryotes [1]. In this pathway, uracil is first reduced to dihydrouracil, catalyzed by dihydropyrimidine reductase (PydA) [2,3]. This de-aromatization of the pyrimidine ring facilitates subsequent hydrolysis by dihydropyrimidinase (PydB) and ureidopropionase (PydC), releasing ammonia and β-alanine for use as nitrogen and / or carbon sources for growth [2–5].

**Figure 1:**
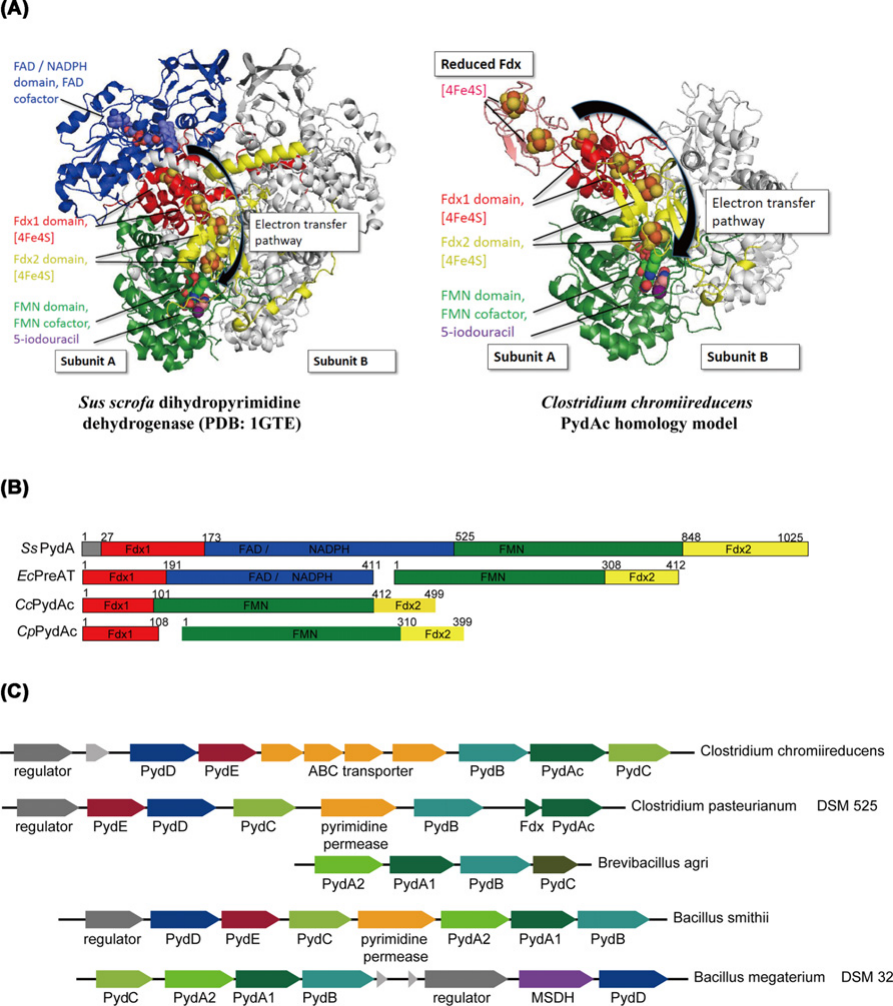
PydAc is a variant of PydA present in anaerobic bacteria (**A**) Comparison of the crystal structure of *Sus scrofa* dihydropyrimidine dehydrogenase (PDB: 1GTE) with a homology model of *Clostridium chromiireducens* PydAc [7]. For the *S. scrofa* enzyme, the homodimer is shown, with the Fdx1 (red), FAD/NADPH (blue) and FMN (green) domains of subunit 1, and the Fdx2 (yellow) domain of subunit 2. Cofactors and a substrate analog are rendered in spheres. The substrate analog 5-ioduracil (purple) is located in the FMN domain. The pathway for electron transfer from the FAD to the FMN site is indicated. For the *C. chromiireducens* enzyme, the same color scheme and rendering is used, showing the lack of the FAD/NADPH domain. The proposed pathway for electron transfer from the putative electron source reduced Fdx to the FMN site is indicated. (**B**) Comparison of the domain structures of PydA variants, including the previously characterized NADP-dependent dihydropyrimidine dehydrogenase from *S. scrofa* (*Ss*PydA), NAD-dependent dihydropyrimidine dehydrogenase from *Escherichia coli* (*Ec*PreAT), and two putative Clostridial ferredoxin-dependent dihydropyrimidine dehydrogenases from *C. chromiireducens* and *C. pasteurianum* (*Cc*PydAc and *Cp*PydAc). (**C**) Pyd gene clusters in bacteria. PydA, dihydropyrimidine dehydrogenase; PydB, dihydropyrimidinase; PydC, ureidopropionase; PydD, β-alanine aminotransferase; PydE, malonic semialdehyde reductase; MSDH, malonic semialdehyde dehydrogenase; PydA1, dihydropyrimidine dehydrogenase Fdx1 and FAD / NADPH domains; PydA2, dihydropyrimidine dehydrogenase FMN and Fdx2 domains; PydAc, Clostridial dihydropyrimidine dehydrogenase homolog.

The only PydA homolog that has been structurally characterized is from *Sus scrofa*, and is a homodimer of 2 × 111 kD [6,7] (Figure 1A, left panel). Each protomer contains five domains (Figure 1B), and multiple cofactors involved in coupling the reduction of uracil to the oxidation of NADPH. The catalytic mechanism of this enzyme involves the rate-limiting oxidation of NADPH occurring in the flavin adenine dinucleotide (FAD) and NADPH domains, followed by the rapid transfer of two electrons to the flavin mononucleotide (FMN) domain, where uracil reduction occurs [7,8]. Electron transfer between the two flavin sites is mediated by two ferredoxin (Fdx) domains, each containing two [4Fe-4S] clusters (Figure 1A, left panel) [7].

The PydA enzymes that have been studied to date, in the context of the Pyd pathway, utilize NADPH as the electron source for uracil reduction. A related enzyme PreAT in *Escherichia coli* utilizes NADH for uracil reduction, and is not involved in the Pyd pathway (*E. coli* does not encode other enzymes in the Pyd pathway such as PydB and PydC, and is unable to use uracil as a nitrogen source for growth) [9]. The physiological function of PreAT remains unknown. While mammalian PydA homologs contain all five domains in a single open reading frame (ORF), many bacterial PydA and PreAT homologs are split into two ORFs, with the first ORF (PydA1, PreA) containing the FAD, NADPH and one Fdx domain (colored blue and red respectively in Figure 1A,B), and the second ORF (PreT, PydA2) containing the FMN and other Fdx domain (colored green and yellow respectively in Figure 1A,B) [3,9].

While investigating variants of Pyd gene clusters in bacteria, we noticed a PydA variant (PydAc), present in Clostridiales bacteria including *Clostridium chromiireducens* and *Clostridium pasteurianum* (Figure 1B,C). PydAc lacks the FAD and NADPH domains, suggesting an inability to catalyze NAD(P)H-dependent uracil reduction. Because the energy metabolism of these strict anaerobic bacteria rely on reduced Fdx as an intermediate [10], we hypothesized that PydAc is a Fdx-dependent dihydropyrimidine dehydrogenase.

Here, we report the biochemical characterization of recombinant PydAc from *C. chromiireducens*. PydAc catalyzed the reduction of uracil with reduced methyl viologen (MV^+^) or reduced Fdx, but not NAD(P)H, as an electron donor. The distribution of PydA variants in metabolically diverse Firmicutes bacteria is discussed.

## Materials and methods

### General

Lysogeny Broth (Luria Broth, LB) medium was purchased from Oxoid Limited (Hampshire, U.K.). *Clostridium chromiireducens* C1 (DSM12136) was purchased from DSMZ (Deutsche Sammlung von mikroorganismen und Zellkulturen GmbH). Methanol and acetonitrile used for liquid chromatography–mass spectrometry (LC–MS) were high-purity solvents from Concord Technology (Minnesota, U.S.A.). Formic acid was purchased from Merck (New Jersey, U.S.A.). Water used in this work was ultrapure deionized water from Millipore Direct-Q. All protein purification chromatographic experiments were performed on an ÄKTA pure or ÄKTA prime plus FPLC machine equipped with appropriate columns. Protein concentrations were calculated from their absorbance at 280 nm, measured using a Nanodrop One (ThermoFisher Scientific, PA, U.S.A.). PydA spectrophotometric activity assays were carried out using a Nanophotometer NP80 Mobile. Anaerobic experiments were conducted in a Lab 2000 glovebox (Etelux, Beijing, China) under an atmosphere of N_2_ with less than 10 ppm O_2_.

### Cloning, expression and purification of PydAc and Fdx

Codon-optimized gene fragments of *C. chromiireducens* PydAc (UniProt accession: A0A399ILH0) and *C. pasteurianum* Fdx (P00195) were synthesized by General Biosystems, Inc. (Anhui, China) and inserted into the modified pET28a vectors HT and HMT, respectively [11]. For recombinant production of PydAc, *E. coli* BL21 (DE3) cells were co-transformed with plasmids HT-PydAc and pTf16 (TaKaRa), and grown in LB supplemented with 50 μg/ml kanamycin, 25 μg/ml chloramphenicol and 0.5 mg/ml L-arabinose. For recombinant production of Fdx with an N-terminal MBP (maltose binding protein) tag, *E. coli* BL21 (DE3) cells were transformed with the plasmid HMT-Fdx, and grown in LB supplemented with 50 μg/ml kanamycin. Both cultures (typically 0.8 L in a 2.6 L flask) were grown at 37°C while being shaken at 220 rpm. When OD_600_ reached ∼0.8, the temperature was decreased to 18°C and isopropyl β-D-1-thiogalactopyranoside (IPTG) was added to a final concentration of 0.3 mM to induce the production of the proteins. After 18 h, cells were harvested by centrifugation (8000 × ***g*** for 15 min at 4°C).

The cell paste was resuspended in lysis buffer (5 ml per g of cell paste) containing 50 mM Tris-HCl, pH 8.0, 1 mM phenylmethanesulfonyl fluoride (PMSF), 0.4 mg/mL lysozyme, 0.03% Triton X-100 and 0.03 mg/ml of DNase I (Roche, Germany). The cell suspension was frozen in a −80°C freezer, and then thawed and incubated at 25°C for 40 min to allow for lysis. A 6% solution of streptomycin sulfate in water was added to a final concentration of 1% to precipitate the DNA. The precipitate was removed by centrifugation (12,000 × ***g*** for 30 min at 4°C). The supernatant was then filtered through a 0.45 μm filter. β-Mercaptoethanol (BME) was added to a final concentration of 5 mM, and the sample was loaded onto a column containing 5 ml TALON Co^2+^-affinity resin (Clontech, California, U.S.A.), pre-equilibrated with buffer A [20 mM Tris-HCl, pH 7.5, 5 mM BME and 0.2 M KCl]. The column was washed with 10 column volumes (CV) of buffer A, and then the protein was eluted with 5 CV of buffer A containing 150 mM imidazole. The eluted protein was precipitated with solid (NH_4_)_2_SO_4_ to 70% saturation and isolated by centrifugation (20,000 × ***g*** for 10 min at 4°C). The pellet was dissolved in 3 ml of buffer A and desalted using a G25 column (GE, U.S.A., thermostat jacket tube XK16/20, packed 15 cm × 2 cm^2^, 30 ml), pre-equilibrated with buffer A. The eluted proteins were concentrated to ∼1.5 ml by ultrafiltration (VIVASPIN TURBO 15 (30,000MWCO), Sartorius, Germany), frozen in aliquots with liquid nitrogen and stored at −80°C until further use. The purified PydAc (*ε*_280_ = 37,360 M^−1^cm^−1^) and MBP-Fdx (*ε*_280_ = 69,330 M^−1^cm^−1^) were examined on a SDS-PAGE gel.

### Reconstitution of the [4Fe-4S] clusters of PydAc and MBP-Fdx

Solutions of PydAc (50 μM) and HMT-Fdx (500 μM) were degassed on a Schlenk line and brought into the glovebox. The reconstitution buffer contained 10 mM dithiotheritol (DTT) and 100 mM Tris-HCl, pH 7.5. For reconstitution of PydAc, a solution of ferrous ammonium sulfate (16 eq.) was added followed by a solution of sodium sulfide (16 eq.). For reconstitution of MBP-Fdx, a solution of ferrous ammonium sulfate (8 eq.) was added followed by a solution of sodium sulfide (8 eq.). Both mixtures were incubated overnight at 4°C in a cooling-heating block (Dry Bath H2O3-100C, Coyote Bioscience, Beijing, China). Solutions of EDTA (16 eq. or 8 eq. respectively) were added, and excess of iron and sulfide removed by repeated concentration with a centrifugal filter unit (1.5 ml YM-30 Amicon, Millipore), and dilution with buffer containing 20 mM Tris-HCl, pH 7.5 and 0.1 M KCl.

### MV^+^-dependent spectrophotometric activity assay for PydAc

MV^2+^ was pre-reduced to MV^+^ with Ti(III) citrate (∼0.8 eq.) in the glovebox, and quantified from its absorbance (*ε*_600_ = 13,700 M^−1^cm^−1^). In a typical assay, the 200 μl reaction mixture contained 0.2 μM reconstituted PydAc, 5 mM uracil, 15 μM FMN, 0.1 mM reduced MV^+^, 0.1 M KCl, and 20 mM of Tris-HCl, pH 7.5. The absorbance at 600 nm was monitored at 5 s intervals using the cuvette mode of a Nanophotometer NP80 Mobile in the glovebox. To determine enzyme dose-dependence, the assay was conducted with 5 mM uracil, and varying concentrations of PydAc. To obtain Michaelis–Menten kinetic parameters, the assay was conducted with 0.2 μM PydAc, and varying concentrations of uracil. Data analysis was conducted using GraphPad Prism6.

### LC-MS detection of dihydrouracil

To detect dihydrouracil formation in the PydAc reaction, a 200 μl reaction mixture containing 20 mM Tris-HCl, pH 7.5, 0.1 M KCl, 15 μM FMN, 1 mM uracil, 10 mM reduced MV^+^ and 5 μM PydAc were incubated at room temperature (RT) for 60 min. Negative controls omitting either enzyme or substrate were also prepared. LC-MS analysis was performed on an Agilent 6420 Triple Quadrupole LC/MS instrument (Agilent Technologies, CA, U.S.A.). The drying gas temperature was maintained at 350°C with a flow rate of 12 L min^–1^ and a nebulizer pressure of 25 psi. LC was carried out on an Agilent ZORBAX SB-C18 column (4.6 × 250 mm, product number 880975-902). A linear gradient of acetonitrile (5-75% in H_2_O) containing 0.1% formic acid and a flow rate of 0.5 ml/min for 39 min were used for elution.

### Fdx-dependent activity assay for PydAc

Reconstituted MBP-Fdx was photo-reduced by incubation with 10 μM acriflavine and 50 mM bicine in ambient light for 90 min in the glovebox [12]. In a typical assay, a 200 μl reaction mixture containing 20 mM Tris-HCl, pH 7.5, 0.1 M KCl, 15 μM FMN, 5 μM PydAc, 1 mM uracil and 1 mM reduced MBP-Fdx was incubated at RT for 60 min in the glovebox. The product was analyzed by LC-MS on a Q Exactive^™^ HF/UltiMate^™^ 3000 RSLCnano (Thermo Fisher Technologies, PA, U.S.A.). The probe heater temperature was maintained at 300°C, electrospray voltage was set at +3800 V, capillary temperature was maintained at 250°C, Sheath Gas was set at 45 arb and Aux Gas was set at 12 arb. For mass spectra acquisition, resolution was 120,000, *m/z* scan range from 50 to 200, and data acquisition mode was full-scan. Uracil and dihydrouracil were detected through positive ion mode. LC was carried out on an Agilent ZORBAX SB-C18 column (4.6 × 250 mm, product number 880975-902) at 25°C. A linear gradient of acetonitrile (5-30% in H_2_O) containing 0.1% formic acid and a flow rate of 0.5 ml/min for 45 min were used for elution.

### Bacterial culture using uracil as the sole nitrogen source

To prepare defined medium, 9 g Na_2_HPO_4_·12H_2_O, 1.5 g KH_2_PO_4_, 0.01 g MnSO_4_·H_2_O, 0.2 g MgSO_4_·7H_2_O, 0.01 g CaCl_2_·2H_2_O, 5 mg Na_2_·EDTA·2H_2_O, 1.5 mg CoCl_2_·6H_2_O, 1 mg ZnCl_2_, 0.1 mg H_3_BO_3_, 0.2 mg CuCl_2_·2H_2_O, 0.1 mg Na_2_MoO_4_·2H_2_O, 0.2 mg NiSO_4_·6H_2_O, 1 mg FeSO_4_ ·7H_2_O, 0.2 mg Na_2_SeO_3_, 0.4 mg Na_2_WO_4_·2H_2_O, 20 μg biotin, 20 μg folic acid, 100 μg pyridoxine-HCl, 50 μg thiamine-HCl, 50 μg riboflavin, 50 μg nicotinic acid, 50 μg D-Ca-pantothenate, 1 μg vitamin B12, 50 μg *p*-aminobenzoic acid, 50 μg lipoic acid, 17.5 μg inositol, 3.75 μg choline, 1.5 g Na-pyruvate and 20 g glucose were dissolved in 1 L distilled water. Either NH_4_Cl or uracil was added as the sole nitrogen source, adjusted to a molar equivalent of 20 mM nitrogen. The medium was then filtered through a sterile 0.22 μm filter.

*Clostridium chromiireducens* C1 was grown in 50 ml of DSM medium 104b in an anaerobic vial at 30°C for 2 days. A 30 μl portion of the culture was then transferred into 3 ml defined medium with different nitrogen sources (20 mM ammonium chloride or 10 mM uracil). Cells were cultivated anaerobically for another 4 days.

### Bioinformatics

The homology model of *C. chromiireducens* PydAc was constructed using Phyre2 [13], with the crystal structure of *S. scrofa* dihydropyrimidine dehydrogenase (PDB: 1GTE) as a template [7] (37% sequence identity between the two proteins).

A list of 3419 bacterial PydA candidates was compiled from the UniProt database [14], identified as containing both dihydroorotate dehydrogenase (InterPro domain IPR005720) [15] and [4Fe-4S] ferredoxin (IPR017896) domains, and containing a PydB candidate in the amidohydrolase family (PF01979) within a 10-ORF window in their genome neighborhood. Genome neighborhood analysis was conducted using the web-based Enzyme Function Initiative Genome Neighborhood Tool [16]. A sequence similarity network (SSN) was constructed using the web-based Enzyme Function Initiative Enzyme Similarity Tool (EFI-EST) [17], and visualized using Cytoscape v3.5 [18]. The *E*-value threshold was adjusted to 10^−140^ (> ∼60% sequence identity is required to draw an edge). The sequence length was restricted to >350 amino acids to exclude partial sequences.

## Results

### Identification of a PydA variant in anaerobic bacteria

Comparison of the domain structure of PydAc from *C. chromiireducens* with that of the previously studied PydA homologs from *S. scrofa* [6,7] and *Brevibacillus agri* [3] show that it contains the FMN domain and two Fdx domains in a single ORF, but lacks the FAD and NADPH domains (Figure 1A,B). A BLAST search did not reveal any homologs of the FAD and NADPH domains in the *C. chromiireducens* genome. A related PydAc variant in *C. pasteurianum* DSM 525 contains the FMN domain and one Fdx domain in one ORF, and a second Fdx domain in a separate ORF (Figure 1B,C). These two PydAc variants are present in several anaerobic bacteria. The absence of the FAD and NADPH domains suggests that PydAc does not use NAD(P)H as a reductant. We hypothesized that PydAc instead uses reduced Fdx as a reductant, because of the prominent role of Fdx in the metabolism of anaerobic bacteria.

### Recombinant production and reconstitution of *C. chromiireducens* PydAc

**Figure 2:**
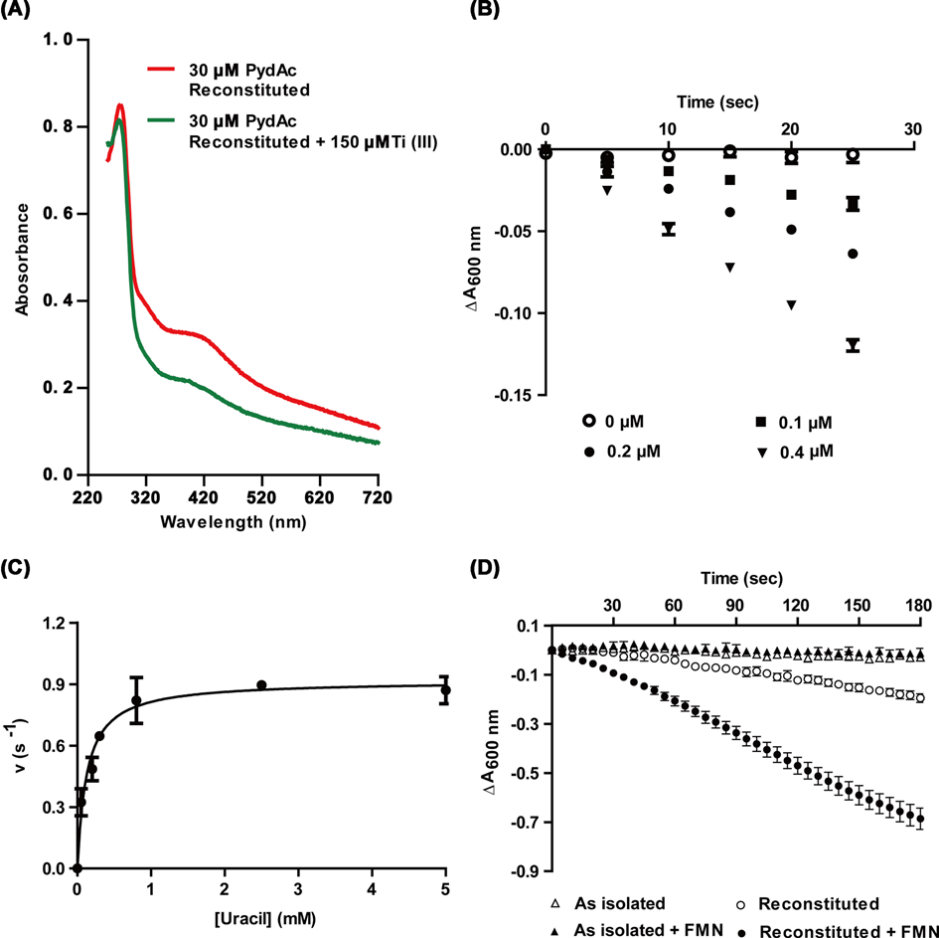
Reconstitution of PydAc [4Fe-4S] clusters and activity assays (**A**) UV–Vis absorption spectra of PydAc. Reconstituted PydAc (30 μM) shows characteristic absorbance of Fe-S cluster at 420 nm (red). Addition of Ti(III) citrate leads to a diminishment of the feature, corresponding to reduction to [4Fe-4S]^1+^ (green). (**B**) Activity assay for PydAc-catalyzed uracil reduction using MV^+^ as the reductant. The assay monitors the decrease in absorbance at 600 nm accompanying the oxidation of MV^+^ to MV^2+^. Assay traces are shown for different concentrations of PydAc, and a linear dependence of reaction rate on enzyme concentration is consistent with an enzyme-catalyzed reaction. (**C**) Michaelis–Menten kinetics of PydAc-catalyzed uracil reduction. The data were fit to the parameters *k*_cat_ = 0.92 ± 0.04 s^−1^, *K*_m_ = 0.13 ± 0.03 mM. (**D**) Comparison of PydAc activities with and without [4Fe-4S] reconstitution and addition of FMN.

To test our hypothesis, *C. chromiireducens* PydAc was recombinantly produced in *E. coli*, and purified to near homogeneity (Supplementary Figure S1). PydAc contains two bacteria Fdx domains, each containing 8 conserved Cys residues for the coordination of two [4Fe-4S] clusters. Purified recombinant PydAc exhibited a brown color, and contained 0.57 ± 0.10 Fe and 0.72 ± 0.10 S per monomer. Anaerobic reconstitution of the PydAc [4Fe-4S] clusters resulted in 12.8 ± 0.2 Fe and 6.9 ± 0.4 S per monomer (out of the theoretical maximum of 16 Fe and 16 S), and typical UV–Vis spectra for a [4Fe-4S] cluster-containing protein (Figure 2A).

### MV^+^-dependent uracil reduction by PydAc

PydAc was assayed for uracil reduction activity using MV^+^ as the electron donor. Anaerobic incubation of purified and reconstituted PydAc in the presence of uracil and MV^+^ resulted in the time-dependent decrease in *A*_600_, corresponding to the oxidation of MV^+^ to MV^2+^ (Figure 2B). No reaction was observed in negative controls omitting PydAc or uracil (Supplementary Figure S2). PydAc exhibited Michaelis–Menten kinetics for uracil reduction (Figure 2C, *k*_cat_ = 0.92 ± 0.04 s^−1^ per PydAc peptide, *K*_m_ = 0.13 ± 0.03 mM). Leaving out the [4Fe-4S] reconstitution step led to abolishment of activity, and omission of FMN from the assay mixture led to a large decrease in activity (Figure 2D). The requirement for FMN addition was not observed for assays of PydA homologs purified from native *S. scrofa* [8] and *E. coli* [9], and may be a consequence of the recombinant PydAc being produced in a predominantly *apo* form.

**Figure 3:**
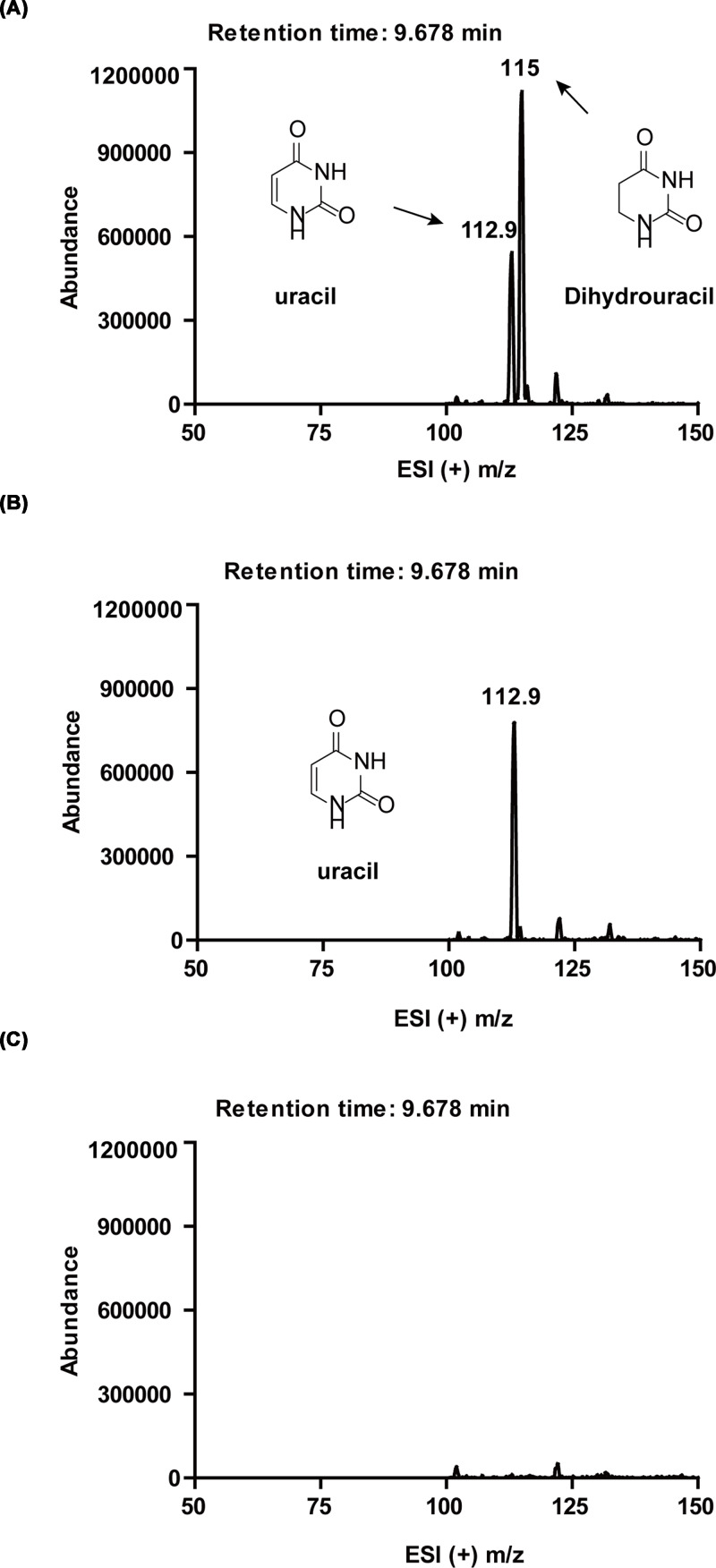
LC-MS analyses of PydAc-catalyzed uracil reduction with MV^+^ as the reductant Both substrate uracil (theoretical *m/z* = 112.9) and product dihydrouracil (theoretical *m/z* = 115) elute at the same retention time, indicated on the mass spectra. (**A**) ESI (+) m/z spectrum of the complete assay, showing presence of both uracil and dihydrouracil. (**B**) ESI (+) m/z spectrum of the negative control omitting PydAc, showing the presence of uracil and absence of dihydrouracil. (**C**) ESI (+) m/z spectrum of the negative control omitting substrate, showing the absence of both uracil and dihydrouracil.

LC-MS analysis of the reaction mixture confirmed the production of dihydrouracil in the complete reaction (Figure 3A). No dihydrouracil was detected in negative controls omitting either enzyme (Figure 3B) or substrate (Figure 3C).

### Fdx-dependent uracil reduction by PydAc

**Figure 4:**
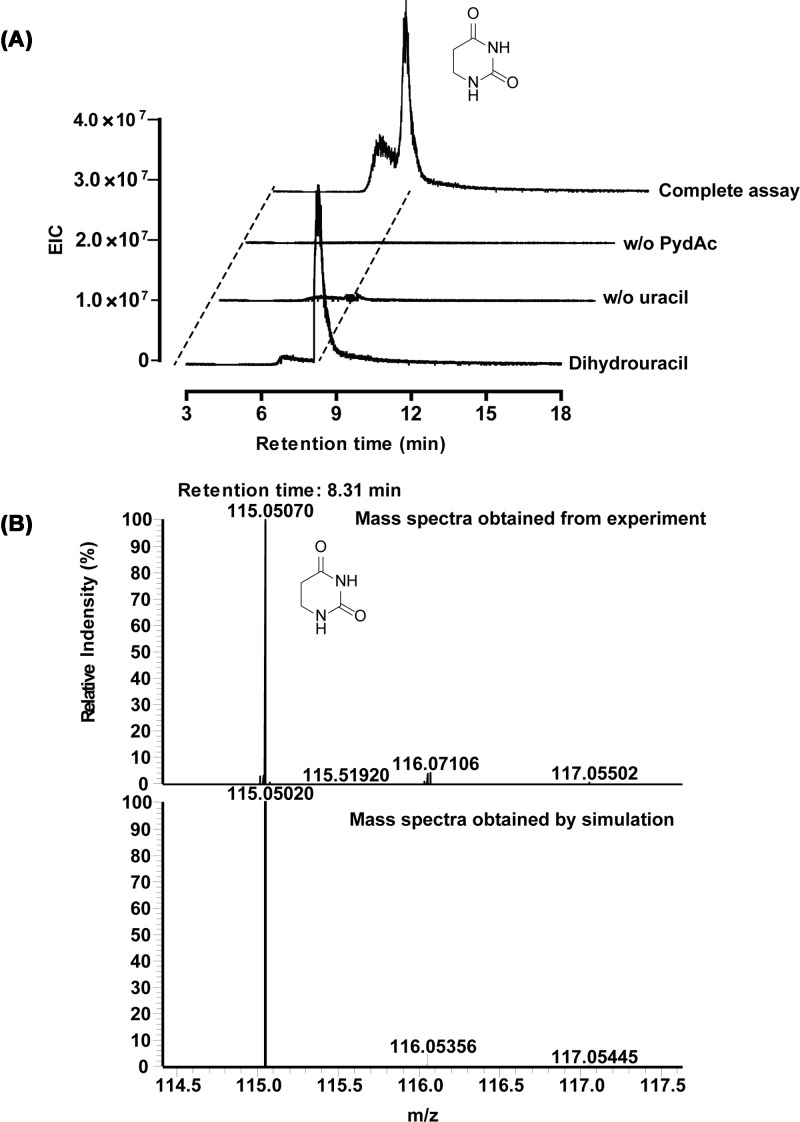
LC-MS analysis of PydAc-catalyzed uracil reduction with ferredoxin as the reductant EIC (*m/z* 115) chromatograms are given to detect the presence of product dihydrouracil (theoretical *m/z* = 115). (**A**) LC-MS elution profiles of authentic standards of dihydrouracil, negative controls omitting ferredoxin and uracil and a complete assay are displayed as labeled. (**B**) ESI (+) *m/z* spectra of the dihydrouracil peak in complete assay and coincidence with theoretical *m/z* of dihydrouracil.

We next tested if PydAc-catalyzed uracil reduction could be supported with photo-reduced Fdx as the reductant, followed by LC-MS analysis. The EIC chromatogram (*m/z* 115) of the complete reaction showed a peak eluting at a retention time of 8.31 min (Figure 4A), corresponding to dihydrouracil, confirmed by ESI-MS (*m/z*) (Figure 4B).

### Uracil as the sole nitrogen source supports growth of *C. chromiireducens*

Next, we investigated the ability of *C. chromiireducens* to use uracil as a nitrogen source. *Clostridium chromiireducens* could grow in defined medium with either ammonium or uracil as the sole nitrogen source (Supplementary Figure S3). No growth was observed in the negative control, in which the nitrogen source was omitted.

### PydAc is present in anaerobic bacteria in the order Clostridiales

**Figure 5:**
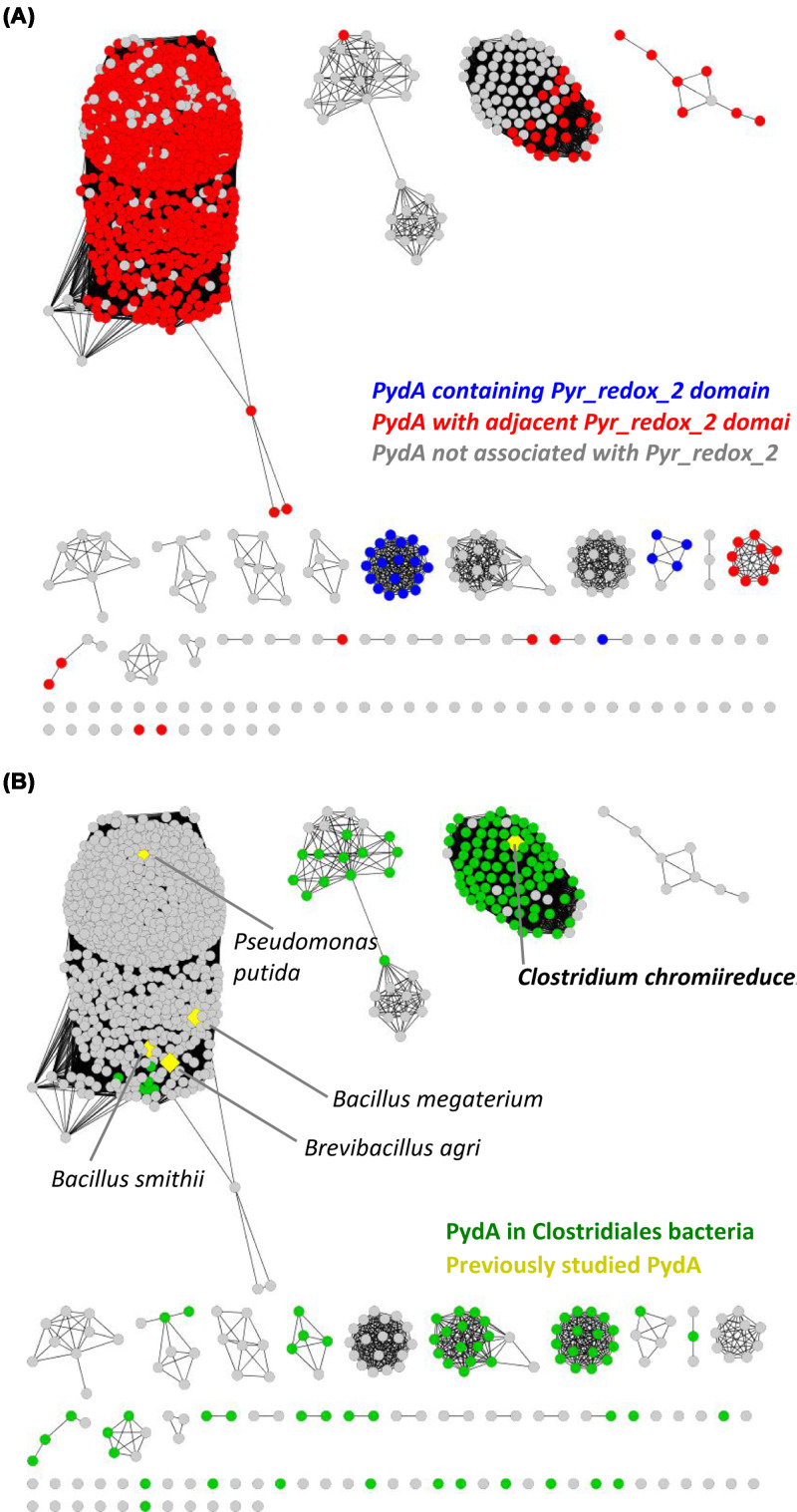
SSN analysis of bacterial PydA homologs (**A**) Putative NAD(P)-dependent dihydropyrimidine dehydrogenases containing a Pyr_redox_2 domain within the same polypeptide (blue) or in an adjacent ORF (red) are respectively colored. (**B**) Sequences from anaerobic bacteria in the order Clostridiales are colored green. Comparison of A and B show that many of the Clostridiales PydA homologs are not associated with a Pyr_redox_2 domain, suggesting that they do not use NAD(P)H as a reductant, and may instead use reduced Fdx as a reductant.

To investigate the occurrence of PydAc in bacteria, a SSN of 3419 candidate PydA sequences was constructed (Figure 5), and the gene clusters were examined for the presence of a pyridine nucleotide oxidoreductase domain (Pyr_redox_2, PF07992), indicative in theory of the ability to use NAD(P)H as a reductant. The majority of the PydA sequences (2781 total) contained a Pyr_redox_2 domain protein in an adjacent ORF (Figure 5A), including several organisms, in which the Pyd pathway was previously studied (*Brevibacillus agri* [3], *Bacillus smithii* [4], *Bacillus megaterium* [5], *Pseudomonas putida* [19]). A further 29 sequences contained a Pyr_redox_2 domain within the same polypeptide.

Among the PydA candidates that were not associated with a Pyr_redox_2 domain, the majority belong to strict anaerobic bacteria in the order Clostridiales (Figure 5B), including *C. chromiireducens* PydAc. The lack of the Pyr_redox_2 domain suggests that these homologs do not use NAD(P)H as a reductant and may, like PydAc, use reduced Fdx as a reductant.

## Discussion

Characterization of PydAc adds to the diversity of the dihydroorotate dehydrogenase (PyrD) family, which includes the pyrimidine biosynthesis enzyme PyrD, and the pyrimidine degradation enzyme PydA. Different classes of PyrD use either NAD^+^, fumarate or quinones as an oxidant for dihydroorotate oxidation [20]. PydA uses NADPH and PreAT uses NADH [9] as a reductant for uracil reduction, while the newly reported PydAc uses reduced Fdx.

Unlike previously characterized PydA homologs, PydAc lacks the NADH/FAD domains. However, it retains the two Fdx domains containing theoretically four [4Fe-4S] clusters, thought to provide a conduit for electron transfer from the FAD to the FMN site, where uracil reduction occurs. We hypothesize that the same route could be taken for electron transfer from reduced Fdx (Figure 1A, right panel).

Our bioinformatics analysis revealed that the PydAc variant most commonly occurs in bacteria in the order Clostridiales. These bacteria use reduced Fdx for their energy metabolism, suggesting the availability of reduced Fdx for the degradative Pyd pathway as well, and highlighting the importance of Fdx-dependent reactions in these strict anaerobic bacteria.

## Supplementary Material

Supplementary Figure S1-S4

## Abbreviations

FAD: flavin adenine dinucleotide
Fdx: ferredoxin
FMN: flavin mononucleotide
Pyd: pyrimidine degradation
PydA: dihydropyrimidine dehydrogenase
PydB: dihydropyrimidinase
PydC: ureidopropionase
