# Supplementary material for: A ferredoxin-dependent dihydropyrimidine dehydrogenase in Clostridium chromiireducens
Source: Biosci Rep. 2020 Jul 2;40(7):BSR20201642. doi: 10.1042/BSR20201642 (PMC7332683; doi:10.1042/BSR20201642)
Supplement: Supplementary Figure S1-S4 [file BSR-2020-1642_supp.pdf]

Figure S1

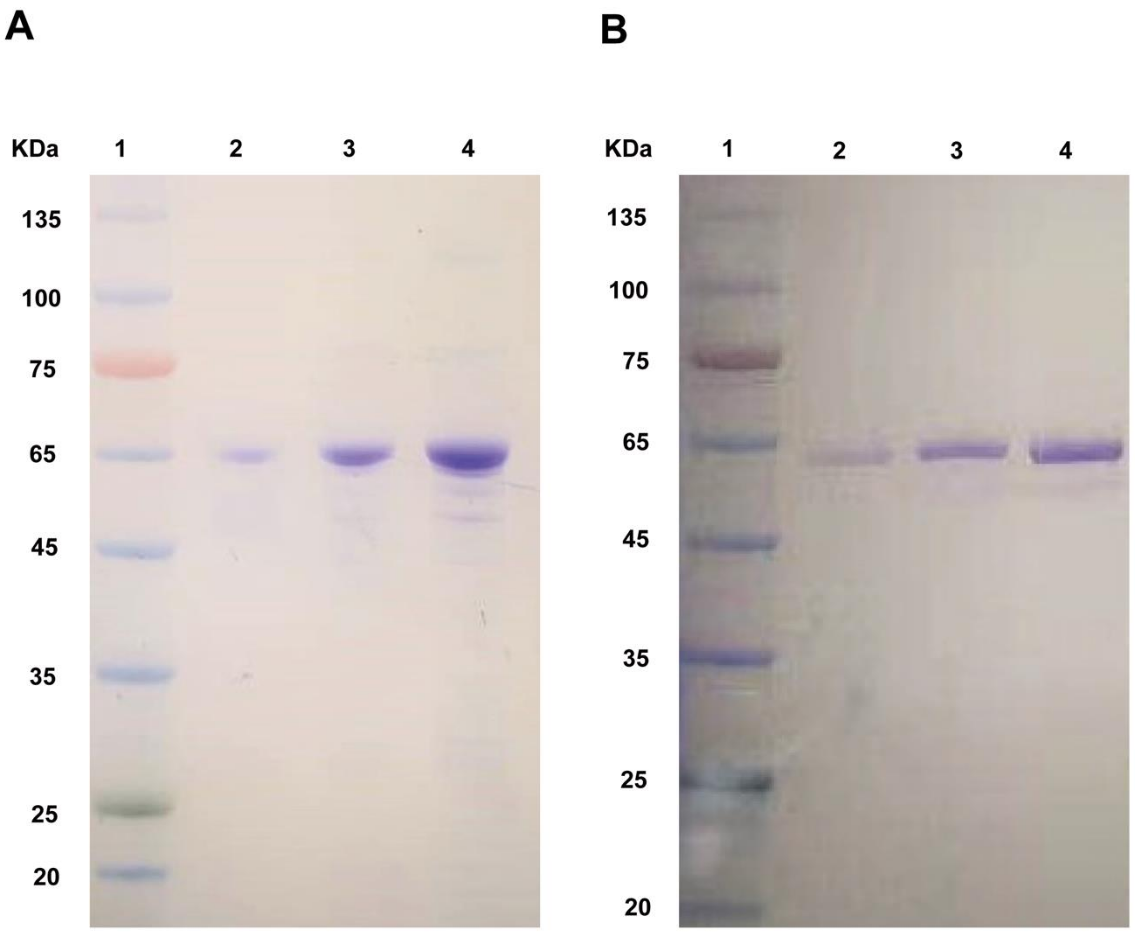
A
B
KDa
1
2
3
4
135
100
75
65
45
35
25
20
KDa
1
2
3
4
135
100
75
65
45
35
25
20

Figure S1. SDS-PAGE analyses of purified of PydAc and MBP-ferredoxin.A) PydAc. B) MBP-ferredoxin. 7% SDS gels with: lane 1, molecular weight marker; and lanes 2-4, 1, 2, 4 μg of PydAc and ferredoxin. The gel was stained with Coomassie Blue.

Figure S2

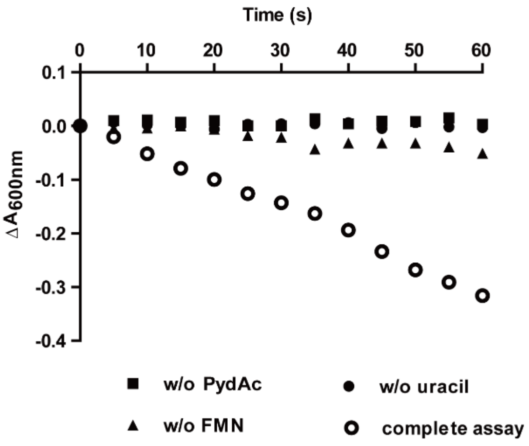
Time (s)
0
10
20
30
40
50
60
0.1
0.0
-0.1
-0.2
-0.3
-0.4
ΔA600nm
w/o PydAc
w/o uracil
w/o FMN
complete assay

Figure S2. Activity assay traces monitoring the decrease in absorbance at 600 nm indicating a consumption of reduced methyl viologen. The complete assay and negative controls omitting the enzyme PydAc, cofactor FMN or substrate uracil are shown.

Figure S3

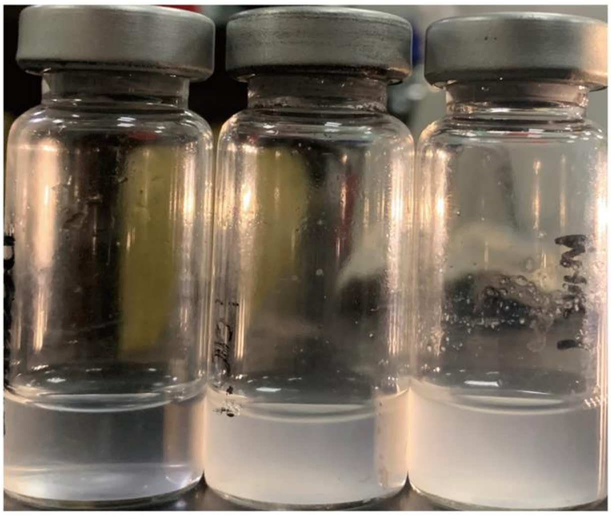

Negative **Uracil** **Ammonium chloride**

Figure S3. Images of *Clostridium chromiireducens* C1 cultures in defined media with uracil or ammonium chloride as the sole nitrogen source. A negative control omitting nitrogen source is included.

Figure S4

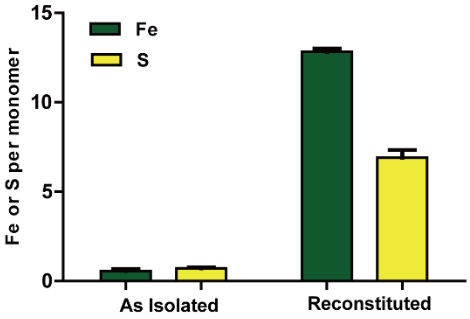

Figure S4. Iron and sulfur content in PydAc. The experiment was performed in triplicate, and the error bars represent standard deviations between the experiments. Green bars represent Fe contents, yellow bars represent S contents.
